# Gender norms and social norms: differences, similarities and why they matter in prevention science

**DOI:** 10.1111/1467-9566.13008

**Published:** 2019-12-13

**Authors:** Beniamino Cislaghi, Lori Heise

**Affiliations:** ^1^ Department of Global Health and Development London School of Hygiene and Tropical Medicine London UK; ^2^ Department of Population, Family, and Reproductive Health Bloomberg School of Public Health Johns Hopkins University Baltimore USA

**Keywords:** social norms, Gender Norms, Low‐ and middle‐income countries, Global Health, Interventions

## Abstract

Two streams of theory and practice on gender equity have begun to elide. The first is work conducted to change *social* norms, particularly using theory that emerged from studies in social psychology. The second is work done on *gender* norms, emerging historically from feminist scholars working to counter gender inequality. As these two streams of work intersect, conceptual clarity is needed to understand differences and similarities between these two traditions. Increased clarity will improve efforts to address harmful norms and practices. In this article, we review similarities and differences between social and gender norms, reviewing the history of the concepts and identifying key tension points of contrast. We identified six areas of comparison that might be helpful for practitioners working for the promotion of global health as they make sense of social and gender norms. We then offer a definition of gender norms for practitioners and researchers working at the intersection between these two theories. Our definition draws from the two different streams of thought of how norms influence people's actions, acknowledging the double nature of gender norms: beliefs nested in people's minds and embedded in institutions that profoundly affect health‐related behaviours and shape differential access to health services.

## Introduction

In recent years, social norms theory has for the first time been applied in low‐ and middle‐income countries (LMIC) to address a variety of health‐related challenges, ranging from adolescent health and female genital cutting, to child marriage and intimate partner violence (Cislaghi and Heise 2019, Gelfand and Jackson 2016, Mackie and Lejeune 2009, Mackie *et al*. 2015). The successful reduction of female genital cutting in Senegal through strategies consistent with social norms theory is a case in point (Mackie 1996, Mackie and Lejeune 2009). Until then, social norm approaches had mostly (albeit not exclusively) been used to reduce unhealthy behaviours in high‐income countries, for example, alcohol consumption (Prentice and Miller 1993, Prestwich *et al*. 2016), smoking (Eisenberg and Forster 2003) or use of recreational drugs (Jiloha 2009, Perkins 2003). The introduction of social norms perspectives into health and international development practice helped focus much needed attention on the ‘social’ reasons why individuals do what they do.

The initial foray of social norms theorists into the global health space paralleled a long‐standing effort on the part of feminists, gender practitioners and women's health advocates to address what this community referred to as ‘inequitable gender norms’. This language evolved in the 1980s and 1990s as part of the larger global project to advance women's rights, transform rigid gender norms and achieve gender equality, as outlined in the global commitments made in the Cairo Plan of Action and the Platform for Action emerging from the UN Conference on Women in Beijing.

Many of the practices now being addressed in low‐ and middle‐income countries, such as female genital cutting (Mackie and Lejeune 2009, Shell‐Duncan *et al*. 2011), child marriage (Chow and Vivalt 2016), women's economic empowerment (Markel *et al*. 2016) or intimate partner violence (Deitch‐Stackhouse *et al*. 2015, McKool *et al*. 2017), are highly gendered. As a result, the emerging stream of work on ‘social norms’ has begun to elide with earlier efforts to address ‘gender norms’, creating some confusion. Significantly, as we describe further below, these two traditions advance very different conceptualisations and understandings of norms and how they operate. Researchers and practitioners in these two communities have yet to develop a common language. Recent initiatives at the intersection between these two streams of work include mentions of ‘gender norms’ (FHI360 and IRH, 2016), ‘gendered social norms’ (Markel *et al*. 2016) and ‘gender‐related social norms’ (Cislaghi *et al*. 2018).

To advance cross‐theoretical work for gender equity and health, there is need to reconcile understanding of norms from the gender equality and social psychology literature. Greater conceptual clarity would facilitate cross‐disciplinary understanding and collaboration in health promotion practice in low‐ and middle‐income countries. For instance, a common language and understanding would assist practitioners more familiar with the social psychology approach to integrate concern for power into their work. Doing so would then help them untangle how power relations between men and women affect the adoption of new positive norms, eventually facilitating more effective health promotion interventions. Similarly, gender specialists might benefit from developing a new understanding from social norms theory about how to shift people's beliefs about what actions are considered acceptable in a given group, potentially widening the toolkit of effective intervention strategies.

The aim of this paper is thus to offer a definition of gender norms for practitioners and researchers working to advance gender equity in health. In the following sections, we look at similarities and differences between traditional conceptualisations of social and gender norms, and at what each field can bring to social improvement efforts. The last section offers a cross‐theoretical definition of gender norms.

## An introduction to social and gender norms

Interest in social norms has diffused across the community of those working to achieve global gender equity (Cislaghi *et al*. 2018, Institute of Reproductive Health, 2016, Lilleston *et al*. 2017, Vaitla *et al*. 2017), who studied gender norms as both a source of and a solution for discriminatory inequalities between men and women (Harper and Marcus 2018). The relative independence of the discourses on social norms and gender norms has resulted in different and fairly separate bodies of scholarship that we present below.

### Social norms

The social sciences have a long‐standing fascination with understanding how humans come to work together and, more specifically, how unwritten rules emerge that affect their actions. Interest in social norms is traceable already in Aristotle, Grotius, Hume and Locke, among others. In the 20th century, anthropologists and sociologists spent considerable time and resources studying how attitudes and practices of the group influence attitudes and practices of individuals (Allport 1924, Bovard 1953b, Durkheim 1951, Mackie *et al*. 2015, Parsons and Shils 1951, Schanck 1932, Sherif 1936, Sherif and Cantrill 1947, Sumner 1907, Thibaut and Kelley 1959). Today, the social norms literature has grown varied and multi‐faceted (Legros and Cislaghi 2019), with multiple definitions – sometimes contradictory – of what social norms are and how they influence behaviour. Generally speaking, social norms are rules of action shared by people in a given society or group; they define what is considered normal and acceptable behaviour for the members of that group (Cislaghi and Heise 2018a). They can influence, for instance, how people dress for a wedding, stand in line when buying something, shake hands when meeting someone, say bless you’ when someone sneezes, offer their seat on the bus to someone older or speak quietly at the library, to cite a few examples. Three features of social norms theory are important to consider as we look to compare this conceptualisation of norms with that dominant in the gender and women's rights community.

First, much literature on social norms conceptualise norms as separate from (and often opposing to) personal attitudes. While personal attitudes are internally motivated judgements about something (Fishbein and Ajzen 1975), social norms, instead, are beliefs about what other people do and approve of. A personal attitude would be ‘I don't like to smoke’, while a social norm would be ‘My friends expect me to smoke’. The difference is important as some people might want one thing, but are pushed by the norm to do the opposite of what they personally lean towards (Miller and McFarland 1987, Prentice and Miller 1996). Interventions using a ‘social norms approach’ historically have leveraged the misalignment between (i) people's individual behaviours and attitudes, and (ii) descriptive and injunctive norms (people's perceptions on others’ behaviours and attitudes) (Bingenheimer 2019). A case in point is interventions to reduce alcohol use in US campuses. These interventions start with a survey measuring prevalence of behaviours, attitudes and norms. For example, a similar intervention could start by measuring (i) how much students drink and approve of those who drink, and (ii) how much they think other students drink and approve of those who drink. When results show a misalignment between behaviour and norms – for example, (i) 20% drink more than one beer on Saturday night, and (ii) 100% think almost everyone drinks more than one beer on Saturday night – the intervention publicises results with the aim to correct similar harmful misperceptions. To do so, traditionally these interventions bear messages such as: ‘80% of students in this university drink only one beer on Saturday night’ (Berkowitz 2010, Perkins and Berkowitz 1986). Similar interventions have been tested in low‐ and middle‐income countries, where a new stream of action suggests that interventions can first change attitudes of a core group of people, then help them become agents of change in their communities, challenging community members’ perceptions of what others in their communities approve of (Cislaghi *et al*. 2019).

Second, various streams in social norms theory posit that norms apply within a ‘reference group’ (Hornsey *et al*. 2003, Smith *et al*. 2007, Terry *et al*. 2000, White *et al*. 2009). That is, different groups of people have different rules. For instance, a young man may feel reluctant to use foul language in front of his family but feel quite comfortable using coarse language when alone with his friends; he adapts his behaviour to the expectations of specific reference groups. Third, while some scholars have suggested that norms regulate only interdependent actions (Goldstein *et al*. 2012, Lapinski and Rimal 2005, Schmidt and Rakoczy Forthcoming), others argue that they inform independent actions as well (Cislaghi and Heise 2018a, Gelfand *et al*. 2006). Independent actions do not require collaboration with others to be carried out (e.g. brushing your teeth at home). Interdependent actions, instead, require coordination between individuals to achieve one's goal (e.g. organising a marriage ceremony) (Van Lange and Balliet 2015). To date, development interventions explicitly based on norms theory have tended to focus on this latter type of action. In the example of female genital cutting in West Africa, for instance, scholars found that – in certain communities (Shell‐Duncan *et al*. 2011) – the cutting was part of a strategy to ensure a daughter's marriageability (Mackie 1996). A mother cannot withdraw her daughter from the tradition of cutting without compromising her daughter's marriage prospects (unless everyone agrees to change the norm of cutting at the same time). Not all theories of norms, however, are exclusively concerned with interdependent actions. Various theories look at how norms influence independent actions, for instance those that focus on socialisation and internalisation of norms (Xenitidou and Edmonds 2014).

### Gender norms

The notion of gender norms emerged in the context of larger debates among academics, practitioners and activists around the nature of gender. Gender as a term was popularised in the 1970s by feminists to distinguish those aspects of male and female roles, behaviours and preferences that were socially constructed rather than a function of biology. The goal was to provide a counterpoint to popular perceptions that male female differences were ‘natural’ and therefore immutable. Feminist sociologists advanced this idea further, arguing that gender is best conceptualised as a social system that apportions resources, roles, power and entitlements according to whether a person or practice is perceived as male or female, masculine or feminine (Ridgeway and Correll 2004). Most existing gender systems are deeply hierarchical, privileging that which is male or masculine over that which is female or feminine (although this need not be the case) (Heise *et al*. 2019, Weber *et al*. 2019).

Norms are but one element of the gender system, along with gender roles, gender socialisation and gendered power relations. In this account, gender norms are the social rules and expectations that keep the gender system intact. The term *gender norms* first entered the health and development lexicon in the last decade of the 20th century, at a time when several international bodies were making a global commitment to promote gender equality (Connell and Pearse 2014). Most early mentions made reference to ‘gendered power imbalances’ between men and women rather than gender norms. But by 2000, the language of gender norms was on the ascendency in academia, with mentions on google scholar rising from 300 between 1985 and 1990 to 16,700 in the decade between 2000 and 2010. Even though much work on gender norms was directed to promoting women's rights and wellbeing, work on men and masculinity likely contributed to this increased interest in gender norms as a construct, with scholarship emerging on how dominant norms of masculinity can result in harm for both men and women (Connell 1993, Connell and Messerschmidt 2005, Courtenay 2000, Evans *et al*. 2011).

Despite the historical interest of gender scholars and activists on gender norms, theoretical work on gender diversified in the 2010s, with the rise of queer studies and transgender activism. Discourse on gender norms and gender as a social system began to coexist with competing understandings of gender as a deeply held psychological sense of oneself as either a man, a woman or something in between. Popular use of the term also changed, as people began to substitute the word gender for sex, losing the important distinction between biology and social construction. While reviewing the entirety of this literature is beyond the scope of this article, Heise *et al*. (2019) recently reviewed how understandings of how gender have diversified over time, with implications for efforts to increase people's health.

The special attention afforded sex and gender with respect to health and behaviour is undoubtedly justified. Gender is a primary frame for social relations (Ridgeway 2009) and an ever‐present part of people's experience of themselves, others and the world (Deaux and Lafrance 1998). Indeed gender is pervasively salient and embedded within relations, power, ideologies and institutions (Connell 1996, 2009). Even compared with race, age and occupation, gender provides the strongest category for differentiation between people (Wood and Eagly 2010). When Haslam and Rothschild (Haslam *et al*. 2000), for example, studied students’ beliefs about categorisation of others, they found that, among 40 categories, male–female categories were thought to be most necessary and immutable. If people understand gender as based on stable properties of the sexes (Prentice and Miller 2006), then gender norms are constructed around primary features present at birth. No other human belief is so constantly salient and primary (Wood and Eagly 2010).

Four core features in the gender norms discourse are relevant for this paper. The first is that gender norms are learned in childhood, from parents and peers, in a process commonly known as socialisation (Bem 1981, Tenenbaum and Leaper 2002) and then reinforced (or contested) in family and the larger social context: through school, the workplace, religion, the media, and other social institutions. The second is that inequitable gender norms reflect and perpetuate inequitable power relations that are often disadvantageous to women (Connell 2014, Lazar 2005). The third contribution of gender theory is the observation that gender norms are embedded in and reproduced through institutions. Policies and regulations, decision‐making processes and biases embodied in how institutions function are a function of a given gender system and reinforce gender norms in the population whose lives intersect with those institutions. Finally, gender norms are produced and reproduced through social interaction, as individuals engage in practices that signify, align or contest various notions of masculinity or maleness and femininity or femaleness (West and Zimmerman 1987).

Thus, two parallel notions of norms, one emerging from feminism and sociological theory and a second emerging from social psychology, are today present in work designed to address gendered practices (such as early marriage; FGC) that have implications for the health and wellbeing of women and girls. In the following section, we compare these two constructs in greater depth and offer a trans‐disciplinary definition of gender norms to help unite the field.

## Key differences between social and gender norms

Social theorists, including anthropologists, sociologists and feminist scholars, tends to conceptualise norms as rules of behaviour at the level of society and institutions (Allport 1933, Durkheim 1951, Parsons and Shils 1951, Pearse and Connell 2015). We find it useful to define this conceptualisation of gender norms as existing in the world *outside* of the individual; they are present when a boy or girl is born, in the world around them. Through various social mechanisms (including socialisation in the family, the media and engagement with institutions), gender norms are enforced, learnt and internalised (Hyde 2014). By contrast, other disciplines, such as social psychology, philosophy and behavioural economics, have tended to define social norms as people's beliefs about what is in the mind of others (Chalub *et al*. 2006, Gintis 2010, Halbesleben *et al*. 2005). Norms thus, in this tradition, exist *inside* the mind. Both approaches include the understanding that the mind and the world influence each other, but each tends to privilege one perspective over the other in their study of norms.

Behind this broad categorisation, further differences exist in the literature on social and gender norms. We identified six areas of comparison that might be helpful for practitioners working for health promotion as they make sense of the social vs. gender norms traditions (summarised in Table 1). These six areas relate to: (i) the type of construct traditionally associated with gender vs. social norms; (ii) the way in which these norms reproduce themselves over time; (iii) the relation between the norms and personal attitudes; (iv) the boundaries within which the norms apply; (v) the processes required for changing them; and (vi) the larger project that animates each tradition.

**Table 1:** Differences between gender norms as understood in the gender literature and social norms as understood in social psychology and behavioural economics

Gender Norms	Social Norms
Gender norms are in the world, embedded in institutions and reproduced by people's actions.	Social norms are in the mind; people's beliefs are shaped by their experiences of other people's actions and manifestations of approval and disapproval.
Gender norms are produced and reproduced through peoples’ actions and enforced by powerholders who benefit from people's compliance with them.	Social norms are equilibria that maintain themselves, not necessarily benefitting anyone.
Gender norms are often studied as shaping people's individual attitudes.	Social norms are often studied as diverging from people's individual attitudes.
People follow the gender norms of their culture, society or group, the boundaries of which are usually blurry.	People follow the social norms of their reference group, the boundaries of which are usually fairly defined.
Changing gender norms requires changing institutions and power dynamics. Often this will happen through conflict and renegotiation of the power equilibrium.	Changing social norms (at its simplest) requires changing people's misperceptions of what others do and approve of in their reference group.
Changing gender norms is a political project that leads to equality between women and men.	Changing social norms is a health‐related project that leads to greater wellbeing for women and men.

In this section, we look at these six points, before offering a trans‐disciplinary working definition of gender norms.

### Nature of norms construct

Traditionally, social theorists looked at gender norms as embodied in institutions (Kenny 2007), media (Gauntlett 2008) and even in the design of cities and buildings (Hayden 1999). As Connell and Pearce (Connell and Pearse 2014) suggested: ‘Gender norms may be embedded in the promotion rules of a government department, in a television station's definition of what information counts as “news”, or an advertising company's habitual imagery of fashionable women’ (p. 7). We thus suggest that gender norms have traditionally been studied as out in the world, shaping people's experience of it and framing their worldviews. We do not mean that work has not been done to understand the socio‐cognitive nature of gender norms; rather, that what emerges as we compare gender norms literature to social norms literature is their political and embedded nature.

Other social scientists (mostly, but not exclusively, social psychologists) have instead been more interested in understanding the cognitive processes that give origin to social norms. They studied social norms as beliefs that originate from observing what people do and like (Bovard 1953a, Griskevicius *et al*. 2008, Mackie *et al*. 2015, Reno *et al*. 1993), and that inhabit the mind of individuals. The distinction has implication for health promotion interventions: if norms are in the mind, changing beliefs will be sufficient for norms to change too. If norms are embedded in laws, policies and institutions, changing gender norms will require a larger project (as we discuss further below).

### Protection and reproduction of normative order

A seminal contribution to the understanding of gender norms comes from the work by West and Zimmerman, who suggested that gender norms are enacted in everyday relations and reproduced through everyday actions (how one talks, dresses, moves, etc.; West and Zimmerman 1987). At the same time, research and practice on gender norms has been traditionally concerned with issues of power and the role of powerholders in protecting existing gender relations (Agarwal 1997, Harper and Marcus 2018). Even though gender norms can be either barriers or facilitators to equality (Connell 2014, Connell and Pearse 2015), health promotion and international development actors tend to look mostly at harmful and discriminatory gender norms (Berkowitz 2003, Elsenbroich and Gilbert 2014a, Harper and Marcus 2018, Mehta and Gopalakrishnan 2007, Pearse and Connell 2015, Sato *et al*. 2015, Temmerman 2015, Usdin *et al*. 2005). As such, a lot of thought has gone into understanding who benefits from unequal gender norms, and the role of powerholders in maintaining the status quo.

Work done on social norms, on the other hand, has been more frequently (albeit not always) less mindful of the role of power relations in sustaining a set of norms, often looking at social norms as resulting from a social equilibrium reached after actors’ trials and errors (Chalub *et al*. 2006, Elsenbroich and Gilbert 2014b, Gintis 2010, Moamin *et al*. 2014, Nowak *et al*. 2015, Ostrom 2014, Prentice 2012, Teraji 2013). Some norms might indeed be less affected by power relations, as, for instance, a norm of wearing seatbelts in the car. Yet, integrating a power analysis into social norms programming would largely benefit for health promotion interventions by drawing attention to powerholders who may resist change.

### Norms and personal attitudes

While scholars and practitioners using social psychological understanding of social norms looked at norms and attitudes mostly in their misalignment (see below), others (most working in the gender norms space) looked at them almost exclusively in their concordance. As mentioned, personal attitudes are one's opinions about something, for instance: ‘I think it would be good for me to carry condoms with me’ (Fishbein and Ajzen 1975). A personal attitude can be aligned with or opposed to an existing social norm (such as, for instance: ‘people disapprove of girls who carry condoms’) (Cislaghi and Heise 2018b). When they are aligned, people both personally think that doing X (carrying condoms in our example above) is good and that X is approved by others. When they are not aligned, people might have a personal preference for doing X, but fear that others will disapprove them for doing so. As a result, individuals might comply with a norm even if it goes against their personal attitude (Chung and Rimal 2016, Cislaghi and Heise 2018a): ‘I would prefer to carry a condom, but I am afraid that people will gossip about me if I did so’. People might thus carry out risky or harmful actions (in this case not carrying condoms or not asking their partners to wear one) that might seem irrational to outsiders, but that make perfect sense to those immersed in the cultural context (Mackie and Lejeune 2009). Some disciplines focus largely on studying people's attitudes when they are discordant from the social norm (specifically when attitudes are protective, and the norm is harmful). Early social norms work on FGC, for instance, studied contexts where people did not want to continue the practice, but did so because they thought it was expected of them. Other disciplines, instead, focus more on the difficult case where attitudes and norms align. This happens when, for instance, people engage in risky behaviour both because they want to and because they think they will profit from doing so (e.g. in terms of belonging or peer approval). Gender norms theory, however, has mostly been applied where norms and attitudes align. Research on gender norms, for instance, has examined how norms of masculinity have shaped people's positive attitudes towards men's authority in the household. Interventions for gender equity have strived to dismantle this normative environment, both by helping people recognise the inequitable status quo and by transforming the inequitable attitudes that have sustained that status quo.

The distinction between concordant and discordant norms and attitudes is important for effective design of health promotion interventions. When attitudes and perceived norms are discordant, interventions often aim to correct people's misperceptions of what others do and/or approve of. When they are concordant, practitioners instead must devise strategies that help shift both personal attitudes towards the practice and the wider norms that support it (at the same time or in a scattered fashion). When attitudes and norms align, there is no misperception of others’ beliefs that can be leveraged, and practitioners would need to address both those norms and attitudes in their change work (Cislaghi and Heise 2018b).

### Norm boundaries

Both the gender and the social norms body of literature posit that different norms apply in different contexts. Yet, a tendency exists in the literature to define the boundaries of ‘context’ in different ways. Even before social norms theory had emerged as field of research and practice, social psychologists and sociologists had suggested that individuals’ behaviours are significantly affected by what others in their group do and think, a theory known as ‘reference group theory’ (Hyman 1960, Merton and Kitt 1950, Nelson 1961, Saxena 1971, Sherif and Cantrill 1947). Empirical research in social norms has often looked at well‐defined reference groups: peer groups, schools, villages, for instance. However, as social psychologist Reid *et al*. (2010) suggested, social norms can exert influence even when the boundaries of ‘the group’ are not clear, as it happens instance in the street, where (he posited) people might align their actions to what they believe to be appropriate in front of strangers (Cialdini *et al*. 1990, Munger and Harris 1989).

The literature on gender norms has more comfortably conceptualised norms as having blurred boundaries, being active within the ethos of a given society or culture. Rather than focussing on a particular group with demographic characteristics (e.g. adolescent girls going into the same school), the gender norms literature looked at how norms manifested themselves within the institutions and narrative of a given culture, and how these norms possibly travel across cultures and places (Amadiume 2015, Brown *et al*. 2017, Oakley 2015).

### Norm change processes

The study of social norms is rich in empirical experiments and interventions that aim to change people's normative beliefs. Clearly, if norms are understood as beliefs, strategies to change norms will target people's beliefs about what others do and approve of. Miller and Prentice (2016) recently conducted a review of what works to change social norms, identifying three key strategies for normative change: (i) personalised normative feedback, where individuals are given feedback on how their behaviour compared to that of their peers or neighbours (used, for instance, to reduce their home energy consumption (Allcott 2011)); (ii) social norms marketing, where individuals are exposed to messages that suggest that most people around them engage in a positive behaviour (used, for instance, to reduce students’ alcohol consumption in US campuses (Prestwich *et al*. 2016)); and (iii) small group discussions, where individuals are invited to identify and correct their misperceptions about what others around them are doing and believe (Steffian 1999).

The gender norms literature, on the other hand, would contend that, when it comes to gender‐related practices, changing people's beliefs is not enough to achieve norm change and eventually people's actions. Change in gender norms would thus require change in institutional policies, people's narrative, power relations and media discourse, to cite but a few examples. Endeavours to achieve gender equity have been either gender transformative (trying to change the gender system by addressing discriminatory power relations) or gender accommodating (compromising with the existing gender system by reaching for lower hanging targets, to avoid harm to the non‐compliers). Often, gender transformative projects include several strategies. The Gender Roles, Equality and Transformation (GREAT) Project, for instance, promoted gender‐equitable attitudes and behaviours among adolescents and their communities in northern Uganda. To do so, GREAT includes community mobilisation for adolescents’ wellbeing, a serial radio drama about young people, and the creation of Village Health Teams (VHTs) that offered youth‐friendly services (Lundgren *et al*. 2018).

### Overarching vision

Finally, most empirical research and action on social norms have traditionally focussed on addressing health‐related issues (P. Yamin, M. Fei, S. Lahlou, and S. Levy, in preparation). These include, for instance: food intake (Vartanian *et al*. 2015), physical activity (Ball *et al*. 2010) and hand washing (Curtis *et al*. 2009). Work on gender norms, however, has been traditionally concerned with issues of gender equality (Heise *et al*. 2019), including: access to employment (Badgett and Folbre 1999, Johnson 2004) or mobility (Balk 1997, Mumtaz and Salway 2005), again just as examples. This is not to say that gender norms efforts have not worked to improve people's (and particularly women's) health – and, in fact, they often did so (Barker *et al*. 2010, Heymann *et al*. 2019, Spencer *et al*. 2015). Rather, we refer to the fact that the work of gender scholars and activists has been traditionally inspired by a vision of gender justice and equality that shaped a value‐based platform to design gender transformative interventions. Like all social movements that aim to change the status quo and the power relations embedded in it, processes or interventions that encourage women and other marginalised groups to challenge norms can result in backlash and harm. Take, for instance, women's access to paid labour and domestic violence: in contexts where the norm dictates that women should not work outside the home, women who challenge the norm and work for pay experience higher risk of experiencing domestic violence (Weber *et al*. 2019). While defying the norm means greater risk of violence and harm, their transgression will eventually help change the norm achieving greater gender equality. As the number of people who transgress the norm increases, more space for others to follow will open‐up, eventually reaching a tipping point and bringing about sustainable change in the status quo. A vision for global gender equality would thus help frame similar interventions as positive, in spite of the potential risk of harm for the population involved (that would obviously need to be mitigated as much as possible).

## A definition of gender norms

Greater intervention effectiveness might come from increasing practitioners’ clarity of the differences between social norms and gender norms. One opportunity to do this is by creating a definition of gender norms that takes into account both intellectual traditions. A starting point might be agreeing on the fact that many social norms are gender norms. People do not simply hold beliefs about what is expected from them, they hold beliefs about what is expected from them because of their sex and socially constructed rules of behaviour assigned to that sex. As men and women comply with these expectations, they adhere to those expectations and beliefs, and contribute to strengthening them in other people. We suggest the following definition of gender norms:

Gender norms are social norms defining acceptable and appropriate actions for women and men in a given group or society. They are embedded in formal and informal institutions, nested in the mind, and produced and reproduced through social interaction. They play a role in shaping women and men's (often unequal) access to resources and freedoms, thus affecting their voice, power and sense of self.

This definition acknowledges the cognitive nature of norms as beliefs, while, at the same time, suggesting that those beliefs are the result of (and shape) very concrete and material realities in which people live and learn. Adopting such a definition also requires accepting that quantitative measures might only partially grasp changes in gender norms. Sometimes measurement is essential and important, researchers should be aware that aspects of gender norms likely remain beyond their reach. For instance, while a part of gender norms might be uncovered by measuring people's expectations of appropriate behaviour for men and women, the institutional aspects or the related power relations might not be captured by these same measures. A multiplicity of methods that include qualitative strategies would thus be better suited to capture how gender norms affect people's lives, and how they shift over time.

## Conclusion

Our paper extends and reinforces the growing body of literature critiquing individualistic approaches to understanding and addressing health inequalities. Several disciplines have provided working frameworks to understand and study people's health‐related behaviour within their socio‐ecological niche (Baum *et al*. 2009, Benatar 2013, Eckersley 2001, Jewkes *et al*. 2015, Marmot *et al*. 2012). The potential for cross‐fertilisation between social norms and gender norms work underscore the need to understand how people's social network (sometimes unwittingly) sustain existing social practices, collectively creating outcomes that individually they do not wish for. In this paper, we looked at the literature on social norms and gender norms, identifying similarities and differences to inform the understanding of practitioners working across these two spaces of thought and action. We found six areas of comparison worth mentioning: (i) the nature of norms construct; (ii) the production and protection of the normative order; (iii) the relation between norms and personal attitudes; (iv) the boundaries of norms; (v) the processes through which norms change; and (vi) the overarching vision that inspired work done by scholars and practitioners in the field of social and gender norms. In the last section, we suggested a definition of gender norms, informed by work done in social norms theory. We offer this definition to scholars and practitioners working to promote global health and gender equality, in the hope it might assist them in their important work.

## Author contributions

BC and LH ideated, drafted and revised the paper.

## Conflicts of interest

The authors declare that they have no conflict of interest.

## Ethics and consent

Not required.

## Funding

BC's time was covered by an anonymous donor.

## Paper context

Gender Norms and Social Norms are two widely used concepts in Global Health Action. At this point in time, these two streams of theory and practice have begun to elide. This paper offers greater conceptual clarity that will help those working on gender and social norms understand differences and similarities between these two traditions. Critical opportunities and challenges emerge from the cross‐fertilisation of these two disciplines, with important practical implications for global health action.
